# Identification of Novel MeCP2 Cancer-Associated Target Genes and Post-Translational Modifications

**DOI:** 10.3389/fonc.2020.576362

**Published:** 2020-12-10

**Authors:** Isabel Castro-Piedras, David Vartak, Monica Sharma, Somnath Pandey, Laura Casas, Deborah Molehin, Fahmida Rasha, Mohamed Fokar, Jacob Nichols, Sharilyn Almodovar, Rakhshanda Layeequr Rahman, Kevin Pruitt

**Affiliations:** ^1^Department of Immunology and Molecular Microbiology, Texas Tech University Health Sciences Center, Lubbock, TX, United States; ^2^Center for Biotechnology & Genomics, Texas Tech University, Lubbock, TX, United States; ^3^Department of Internal Medicine, Texas Tech University, Lubbock, TX, United States; ^4^Department of Surgery, Texas Tech University, Lubbock, TX, United States

**Keywords:** MeCP2, breast cancer, post-translational modification, ChIP-Seq analysis, transcriptional regulation

## Abstract

Abnormal regulation of DNA methylation and its readers has been associated with a wide range of cellular dysfunction. Disruption of the normal function of DNA methylation readers contributes to cancer progression, neurodevelopmental disorders, autoimmune disease and other pathologies. One reader of DNA methylation known to be especially important is MeCP2. It acts a bridge and connects DNA methylation with histone modifications and regulates many gene targets contributing to various diseases; however, much remains unknown about how it contributes to cancer malignancy. We and others previously described novel MeCP2 post-translational regulation. We set out to test the hypothesis that MeCP2 would regulate novel genes linked with tumorigenesis and that MeCP2 is subject to additional post-translational regulation not previously identified. Herein we report novel genes bound and regulated by MeCP2 through MeCP2 ChIP-seq and RNA-seq analyses in two breast cancer cell lines representing different breast cancer subtypes. Through genomics analyses, we localize MeCP2 to novel gene targets and further define the full range of gene targets within breast cancer cell lines. We also further examine the scope of clinical and pre-clinical lysine deacetylase inhibitors (KDACi) that regulate MeCP2 post-translationally. Through proteomics analyses, we identify many additional novel acetylation sites, nine of which are mutated in Rett Syndrome. Our study provides important new insight into downstream targets of MeCP2 and provide the first comprehensive map of novel sites of acetylation associated with both pre-clinical and FDA-approved KDACi used in the clinic. This report examines a critical reader of DNA methylation and has important implications for understanding MeCP2 regulation in cancer models and identifying novel molecular targets associated with epigenetic therapies.

## Introduction

Epigenetic dysregulation involving mutations or abnormal expression of DNA methylation readers has been associated with a broad spectrum of disorders that range from Rett Syndrome to human cancers (1–7), and alterations in both the writing and reading of epigenetic marks have been linked with tumor progression at every stage (8–12). Aberrant DNA methylation not only promotes disease progression but is targeted *via* therapeutics applied in the clinic (13–15). Because of the prevalence of abnormal epigenetic changes in tumor progression (16–22), exploitation of this property led to FDA approved “epigenetic” therapies (23, 24). Interestingly, DNA methylation readers, such as methyl-CpG-binding protein 2 (MeCP2), bridge DNA methylation and histone modifications by binding to methylated DNA and recruiting co-repressor proteins (25–28). While both normal and abnormal DNA methylation is read by MeCP2, much remains unknown about its role and regulation in cancer-associated pathologies. MeCP2 was shown early on to have an affinity for 5-methylcytosine in the context of methylated CG dinucleotides (mCG) (29, 30) and methylated CH (mCH), where H = A/C/T. MeCP2 binds methylated cytosine (31–33) and shows selectivity for mCG sequences with adjacent A/T sequences (34). However, it also binds to hydoxymethylated cytosine (31, 32, 35–37). While more investigation is needed, MeCP2 binding to mCH has been primarily noted on mCA (31–33, 35, 36, 38). Studies have also shown that MeCP2 binding in mouse brain is proportional to mCAC + mCG density wherein transcription is sensitive to MeCP2 occupancy (38). Additionally, MeCP2 regulates tumor suppressor genes (TSG) silencing, and serves as a critical bridge for histone methyltransferases (HMTs) (25), histone deacetylases (HDACs) (26, 28, 39), and other proteins that bind modified histones or that mediate nucleosome remodeling (27, 40, 41). Moreover, MeCP2 has been reported to be amplified in diverse cancer including human triple-negative breast cancers (TNBC), and it activates growth factor pathways targeted by activated Ras, MAPK and PI3K pathways (42). Novel interacting protein partners and gene targets in brain tissue have also been identified (43). These are the types of enigmatic and versatile properties of MeCP2 that have contributed to long-standing knowledge gaps. We previously reported that inhibition of SIRT1 triggers acetylation of endogenous MeCP2 at lysine (K171), a site that regulates MeCP2 interaction with HDAC1 and ATRX (44). These findings demonstrated that MeCP2 post-translational modifications (PTMs) can critically impact its function, yet few PTMs have been mapped despite the potential that they might affect substrate specificity (35, 45). This knowledge gap is especially important given reports demonstrating unique characteristics of MeCP2 domains in determining binding specificity (46, 47) and the impact of MeCP2 on chromatin-dependent regulation of epigenetic writers (48). In the present study, we have identified additional novel PTMs across the length of MeCP2 and target genes in cancer models. Our findings provide new insight on the versatile role of MeCP2 which is known to be critical in regulating gene imprinting (49), transcriptional activation and repression (50) in disparate conditions that range from autism to cancer (4, 51, 52).

## Materials and Methods

### Cell Lines

MDA-MB-468 (HTB-132), MCF7 (HTB-22), MCF10A (CRL-10317), MCF12F (CRL-10783), PC3 (CRL-1435), T47D (HTB-133), BT549 (HTB-122), and MDA-MB 231 (HTB-26) cell lines used in this manuscript were purchased from ATCC which utilizes STR technology for cell authentication. Cells were used at a low passage (<20) within 6 months or less after receipt or resuscitation. MDA-MB-468, T47D, and BT549 cells were cultured in RPMI 1640 (Gibco). MCF10A and MCF12F were cultured in HuMEC medium supplemented with HuMEC supplement kit (Gibco). PC3 cells were cultured in ATCC formulated F-12K media (ATCC). MCF7 cells were propagated in MEM while MDA-MB-231 cells were cultured in DMEM (Gibco). T47D and MCF7 cells were cultured in media supplemented with 0.1% insulin (Sigma). All cells were grown in culture media supplemented with 1% pen-strep and 10% fetal bovine serum from GIBCO at 37 ° in 5% CO_2_.

### Plasmids

pCDNA3.1 (−) was used as the backbone and Hemagglutinin (HA)-tagged-MeCP2-WT-pCDNA3.1 (−) (encoding MeCP2 e2 isoform), HA-tagged-K135Q-MeCP2-pCDNA3.1 (−) and HA-tagged-K135R-MeCP2-pCDNA3.1 (−) were generated using outward PCR method.

### Bioluminescent MDA-MB-468 Cells

The pGL4.50[*luc2*/CMV/Hygro] plasmid (E1310) which encodes the luciferase reporter gene *luc2* (*Photinus pyralis*) was purchased from Promega. MDA-MB-468 cells were plated in a 6-well plate (Genesee) at the seeding density of 2 × 10^5^ cells in order to reach 60% confluency at the time of transfection. Cells were transfected with 1 µg of the pGL4.50[*luc2*/CMV/Hygro] plasmid for 48 h. Stable transfectants were selected with 0.5 mg/ml hygromycin (Sigma H3274-100MG)-containing media which was replaced every 3–4 days until total selection was achieved. Bioluminescence was confirmed by *In Vivo* Imaging System (IVIS) in the presence of luciferin substrate (Promega VivoGlo™ Luciferin, *In Vivo* Grade P1041).

### MeCP2 Stable Knock-Down and Clonal Selection

MDA-MB-468 cells stably expressing pGL4.50[*luc2*/CMV/Hygro] plasmid (Promega E1310) were plated at the seeding density 2 × 10^5^ cells in order to reach 60% confluency at the time of transduction 48 h prior to infection and then infected with pLKO.1-puro based shRNA MISSION lentiviral transduction particles purchased from Sigma for MeCP2 (TRCN0000330971, TRCN0000330972) and Non-Targeting shRN control transduction particles (SHC002V). The transduction was enhanced with 5 μg/ml polybrene (Sigma Millipore) and 2× multiplicity of infection (MOI) viral particles was added to the media. After 24 h, culture media was replaced with fresh media for 2 days. Stable clones were selected with 6 μg/ml puromycin-containing media which was replaced every 3-4 days until selection was achieved and knockdown confirmed by Western blots and qPCR.

### MeCP2 Stable Overexpression and Clonal Selection

MDA-MB-468 cells stably expressing pGL4.50[*luc2*/CMV/Hygro] plasmid with >90% knocked down of endogenous MeCP2 were plated at the seeding density 2 × 10^5^ cells in order to reach 60% confluency at the time of transfection, 48 h prior to transfection and then transfected with 1 ug of the pCDNA3.1 (−) backbone, Hemagglutinin (HA)-tagged-MeCP2-WT (encoding MeCP2 e2 isoform), HA-tagged-K135Q-MeCP2 and HA-tagged-K135R-MeCP2 plasmids. The G418 disulfate salt solution (Sigma G8168-10ML) selection was started 48 h after transfection at a concentration of 0.4 mg/ml, and the G418 containing media was replaced every 3–4 days until total selection was achieved and overexpression confirmed by Western blots.

### RT-PCR and qPCR

Total RNA was isolated using the Aurum™ Total RNA Mini Kit (Bio Rad) and 2 µg of RNA was used to produce cDNA *via* the SuperScript^®^ III First-Strand Synthesis System for RT-PCR (Invitrogen). Intron-spanning primers designed for gene expression analysis are summarized in **Table 1**. All primers were validated by end-point PCR (RT-PCR), a minus reverse transcription control (−RT control) was included in all RT-PCR experiments. Equal amount of synthesized cDNA was used for qPCR using the Power UP SYBR Green (Thermofisher Scientific, A25778) and the CFX96 Real-Time System C1000 Touch Thermal Cycler (Bio Rad). β-actin gene expression was used as endogenous control for mRNA quantification, as is not a MeCp2 target gene in both cell lines studied and its expression didn’t change after the depletion of MeCP2 in RNA-seq analysis.

**Table 1:** qPCR Primers used in the current study.

	Forward primer	Reverse Primer
**qPCR**		
CDH1	TGC CCA GAA AAT GAA AAA GG	GTG TAT GTG GCA ATG CGT TC
EGFR	CTT CCT CCC AGT GCC TGA ATA	CTC CGT GGT CAT GCT CCA AT
HDAC1	CGA GAC GGG ATT GAT GAC GA	ACT TGG CGT GTC CTT TGA TAG T
HIPK3	AAA AGG ACG ATC TGC CCC TG	TCC AAA GTG CTG AAC CTG ACT
IL6	CCA GGA GAA GAT TCC AAA GAT	GGA AGG TTC AGG TTG TTT TCT G
KDM3A	TCA CAG GAG CCA CAG TAG GA	AGA TCA TCA AAC CTG GAA GGC A
KDM3B	CCC ACA CCA GGT TCA CAA TCT A	GCC AAC CGC ATC TTT CAC TG
KMT2B	CTC CGG AAG TGC ACC TTT GA	CCG TGG ATG GCT GAT CTG TAG
LANCL2	GGC AGC AAA AGT GGA CCA AG	CTC GCT GCC AAA TCA CAT CG
MALAT1	GCC TGG AAG CTG AAA AAC GG	TGG AA AAC GCC TCA ATC CCA
MAP1B	GAG ATG CTG CCA ATG CCt CT	CAG GGT CAT TCC CAC TCA CC
MECP2	CCC ATC AAC ACG GAA GAA AAG T	GCA GGG TGG GGT CAT CAT AC
MRPS17	TTG GCG GAG GTG ACC AAA	ACA TGC TTT GCT CGT GGA AC
NEAT1	CCC TGT GCT TCC GAC TTC AT	CCC TGG CCT AGT GGA AAT GG
NUPR2	AGC TTT ACG ACT GCC TGG AC	CTT CGA ACA GGT CCT TCG GT
PSPH	CGG CAT AAG GGA GCT GGT AA	GGC TGC GTC TCA TCA AAA CC
SRA1	AGC CCA CAA GTT TCC CAG TC	GGC TTG AAA GCT CTT GCA CC

### Western Blots

Protein extracts were generated using RIPA lysis buffer supplemented with protease inhibitor cocktail (Thermo Fisher Scientific). The protein concentration was measured with the BCA method. Approximately 50 μg of protein from each sample was loaded on NuPAGE™ 4–12% Bis-Tris Protein Gels (Thermo Fisher Scientific) and run at 175 V constant voltage. A constant voltage of 30 V was used for protein transfer onto polyvinylidene fluoride (PVDF) membranes (Millipore-Sigma). Blots were probed with rabbit anti-MeCP2 antibodies (1:1,000; Cell Signaling) and mouse anti-HA antibodies (1:2,000; Santa Cruz) overnight at 4°C. After three washes with tris-buffered saline and polysorbate 20 (TBST; Fisher scientific), blots were then incubated with anti-rabbit HRP conjugate secondary antibody (1:5,000) and anti-mouse HRP conjugate at room temperature for 1 h. After washing three times, chemiluminescence (Pierce ECL Western blotting substrate: Thermofisher Scientific, A25778) was then used to visualize protein bands. β-actin antibody (1:10,000; Santa Cruz) was used as control.

### Immunofluorescence

About 1 × 10^5^ cells were plated on coverslips 48 h prior and they were washed with PBS and then fixed with 4% paraformaldehyde for 15 min. After washing with PBS they were then permeabilized with 0.2% Triton X-100 for 20 min and blocked with 5% BSA for 30 min. Following that, cells were incubated with primary antibodies MeCP2 (Cell Signaling, 3456S), or HA (Cell Signaling, 3724S) for 1 h at room temperature, washed with PBS three times and then incubated in the dark with Phalloidin 568 and secondary antibodies ALEXA-488 goat anti rabbit conjugate for 1 h at room temperature. After three PBS washes the coverslips were mounted with ProLong^®^ Gold Antifade Mountant with DAPI (Thermo Fisher Scientific), the slides were allowed to cure for 48 h and then examined under the Nikon T-1E scanning confocal microscope, with a 60× objective, and analyzed with NIS software.

### Chromatin Immunoprecipitation (ChIP)

Cells were plated for 72 h and the media was changed 24 h prior to the experiment. The cells were subjected to 1% formaldehyde cross-linking (Sigma) for 8 min at room temperature. The cross-linking reaction was quenched by adding glycine (Sigma) to a final concentration of 0.125 M for 5 min at room temperature. The medium was then removed and cells were washed twice with cold PBS containing protease inhibitor cocktail (Thermo Fisher Scientific). Cells were scraped in PBS and pelleted. Pellet was resuspended in SDS Lysis buffer (50 mM Tris–HCl pH 8.0, 10mM 0.5M EDTA, and 1% SDS) with protease inhibitor cocktail and sonicated in a Diagenode Bioruptor 300 sonicator. Sonication conditions involved 20 cycles (30” ON/30” OFF) for MDA-MB-468 and 25 cycles for MCF7 cells. Sonication was evaluated for every experiment in a 1% agarose gel and chromatin fragments showed a distribution in the 100–800 bp range. The soluble chromatin fraction was collected and incubated for 2 h at 4°C with either MeCP2 (Sigma, M9317), or rabbit IgG (Sigma I5006). Dynabeads Protein A (Invitrogen) were washed and then added to the chromatin-antibody mixture and mutated for 2 h at 4°C. Beads were washed with a low salt wash buffer (0.1% SDS, 1% Triton X-100, 2 mM EDTA, 20 mM Tris–HCl pH 8.1, and 150 mM NaCl), a high salt wash buffer (0.1% SDS, 1% Triton X-100, 2 mM EDTA, 20 mM Tris–HCl pH 8.1, and 500 mM NaCl), and TE (1 mM EDTA and 10 mM Tris–HCl pH 8). Reverse-crosslinking was performed at 65°C overnight followed by treatment with RNaseA (Invitrogen) for 2 h at 37°C and proteinase K (Invitrogen) at 55°C for 2 h. The chromatin was eluted and purified using Qiaquick PCR purification kit (Qiagen) and subjected to ChIP-PCR to evaluate the occupancy of MeCP2 along the promoters of target genes. The primers listed in **Table 2** were used for analysis.

**Table 2:** ChIP-PCR Primers used in the current study.

	Forward primer	Reverse Primer
**ChIP-PCR**		
ACOT2	GGT TTT GCT GTG ATG GCT CT	AAG GTA GTG TGT GTT GGG GTA G
AP1M2	ACA GAG ATG TGC GGT GCA A	TTC CAC CCT CAG CCA TTG AT
AQP1	ACT TCA GCA ACC ACT GGG TAG	ATT TCC TGT CCT CTG GCT GTC
ATG4D	TGT ACC GTG GGC TTC TAT GC	CAC CTT TTC AGG GGT GGA CA
CCT6A	TTG GTT CAT TGC GAC TAC CA	TCA CTT GAG GCC AGG AGT TT
CHCHD2	AGT AAT GGC GTG ACC CAA TGT	TGG TTG GAA TTG GGA ACT TGA TG
DKK1	ATT GGC AGG AAC AGG ATG TGT	GAG TGG AAT GAG GAA GGA TTT GT
DLX1	GGT CTT CAT TTG TTG CTC GC	AAT CCT TCC TGC GCC CTA AT
DNMT1	CAA AAG GGG AAC CTT GTT CA	CCT GGG AGG AAG AAA TAG GG
DUX4	TTG CCA TTT GTT CAC TCT GC	TGA TCC TGG GGT TGA AAG TC
EGFR	CCT TGG CAC CTT TCT ACT GC	TGG AAA ATC GGC TTC AAA AC
EIF3G	ATT GGC TCA GGG CTA TTG GT	GGC TTT GCC TTC ATC AGC TT
EYA2	ATG AAG AAC AAG CCC CCG AG	GGG GCT GAG GGT GCA TAA AT
FBXL7	ATC CAG AGA GGT GCG TTT GG	GAG TGG GTG CAG ACA GAC AA
GBAS	CCT CTT GGG AGG TCA ACA AA	GCA TAC GTC ATC TTG CAT GG
GHSR	TTC AGA GTG GAG AGC TTT CTC TGG	TGA GCT GAC TAT CTC TCT CCC A
GRK7	AAT GGT TGC TGG ACG AAC AC	ATC CCT GTG GAC AAT ACT GGT G
H2AFB2	AGA TAG CAC ACT CAA CGC CC	CTT GGC CGT CAG GTA CTC AA
HDAC1	CTG AGC TAA ATC AGC ACC CG	CCT CCC ACT GCC CTA CAT AGA
HIPK3	TCC TTC CCG ACC TCA CAC A	ACT GGC ACT CTT TCA TGG TGG
HIST1H4F	TGT TTG TCT TCG ATC ATG TCT GG	GGC GTC CCG TAT CAC ATT CT
ICAM1	ATT GTC CGG GAA ACT GGA CG	ACA ACA GGC GGT GAG GAT TG
ICAM3	CAT GGT CCA GTG GGA AAG GT	ATA GGC TTG TAC GCC ATC CC
ICAM5	ACT AGA CGG AAG TGG GAC AGA	GTC AAG TTT CCA CCA CGC AG
ID2	GAG CTG TGC GTG AAA TTG CT	ACC GCT TAT TCA GCC ACA CA
IL6	AGC ATC CCT CCA CTG CAA AG	GTG CCC ATG CTA CAT TTG CC
ILF3	AGG CAG CTA CTC CTA CTC GAA	CAC CAC TTG TCC TCC TCC TAA
JARID2	ACG AGT GTA TGG GTG AGT GC	CCA TTG CAG CCA TTT GTC CC
KDM1A	AAG CCA ACG GAC AAG CTG TA	ACA TCA CAT CAT CTC TAC CCT CA
KDM1B	AGT TTG GAA AAC CTG CAA CAC T	AGA GTA GGT GAT TTC GCT GGG
KDM2A	TGC TTC TCA ATG TGC TCT CCA	GCC AGG CTG AAA ACA CTT ACT T
KDM3B	AAC TCC TTT GCT CTC AGC GT	TCC AAA TCT TAC CTC CCC GTC
KDM4A	GGG TCA AAG CAC TTG GGG AT	GCT TCA CAG AGC AAC AAG GC
KMT2A	ACC ACC ATG TGA CTA TTG GAC TT	ACA GCT CTT ACA GCG AAC ACA
KMT2B	AAC CCC ACC CAT TTC CCT GTT	TGG GAG GCC AGG AAG TTG AA
KRI1	TGA TAA ATG CGG GGG TCC TT	TCC ATC CTA ATC CCT ACG CTG A
LANCL2	CCA TTA ACT TGG GAG GCT GA	GGA CTG CAA TGT CAC CAA TG
METTL7A	GCT CTG TGG ATG TGG TGG TC	CTC ACA CCC TTT CAC TCA CCG
MRPS17	TAG GTG CCA AGG ATG GTT TC	CTC CCA AAG GTC AAG GAT CA
NUPR1L	AAA GCC TGC GGA ACT TCA TA	GGA TGG TTT CGA TCT CCT GA
OXCT2	TTG ATG TCG TCC ACC GTC AG	GAC CTG GCG AAC TGG ATG AT
P2RY11	CTG GTG GTT GAG TTC CTG GT	GCT GAT GCA GGT GAT GAA GA
PPAN-P2RY11	GAC ACT GTC TCT CCC CAC AGA	CCC AAT CTG GGG CGT TCA ATC
PSPH	GTG CTT GAA GGT GGG TAG GA	CTG TGG ATT CTG CAA GAG CA
SEC61G	CCT GGG TTC AAG CAA TTC TG	CAC CCG AGG TCA GGA GTT TA
SIRT1	AAG AAA GGC AGT CGG ACC AT	GCT GAC CTA CAG TAA GCA CTC A
SIRT5	AAC CAC AGA CCT GCC TGA GT	TCC CTC TCC CAT CAG GGT AT
SLC44A2	ACC CTA CTT CAT GTC GCC C	TCA TGC ACC CCC AGT CTA CAT
SUMF2	CAA CAC AGA CCC CCA TCT CT	CAT GGC TCA CTA CAG CCT CA
TCEB3C	CTC AGA AAT CGC CTC CTG TC	GAG AGT GCT TCT GGG TTT GC
TCEB3CL2	CTC AGA AAT CGC CTC CTG TC	GGA GAG TGC TTC TGG GTT TG
VOPP1	TTC CAC AGC ACT CCT CAC AG	GAA TGA GGC AGC AGA AGT CC
VSTM2A	AAG GTT GGA TGG GTT TTT CC	ACA CTG GCA AAT TCC GTT TC
WNT3A	GGC ATG GGG AGG TAT GCA AT	GAA TCT GGC CGT GTG CTT TG
WT1	GCT TGA ATG AGT GGT TGG GGA	ACC GCT GAC ACT GTG CTT CTC
ZNF154	TCT CCA GCA TCA CAT CAC GG	TCC TCT CAG TTG GGG AGC TT
ZNF713	AGT CAC AAA AAT CCA GAG CCC A	AGA ACA GGC AGG AAT CCA TGA

### ChIP Sequencing

For ChIP-Seq experiments, ChIP DNA was prepared as described above library preparation was followed by high throughput sequencing with Illumina Hi-seq 2000 at GENEWIZ Corporation.

### RNA Sequencing

RNA was prepared as described above, and library preparation and sequencing were performed at Center for Biotechnology & Genomics of Texas Tech University. RNA quality was determined using RNA Screen Tpe (Agilent). Ribosomal RNA depletion was achieved using NEB Next rRNA Depletion Kit (Human/Mouse/Rat) (NEB # E6310X). RNA fragmentation, double stranded cDNA and adaptor ligation was generated using NEBNext Ultra II Directional RNA Library Prep according to the manufacturer’s protocol (NEB # E7760L). PCR enriched libraries were quantified by Qubit and equimolar indexed libraries (different samples had different indexes for multiplexing) were pooled. Pooled libraries were quantitatively checked using the Agilent Tapestation 2200 and quantified using Qubit. The libraries were then diluted to 200 pM and spiked with 2% phiX libraries (Illumina control). The transcriptome sequencing was performed on the barcoded stranded RNA-Seq libraries using Illumina NovaSeq 6000 SP flow cell, paired-end reads (2 × 50 bp).

### ChIP Sequencing and RNA Sequencing Data Analysis

For ChIP-Seq analysis, the FASTQ files were analyzed using DNASTAR’s Laser Gene software. MEME-ChIP was used to analyze MeCP2 binding motifs and TOMTOM to identify if those motifs were similar to known consensus sequences using the MEME Suite Programs http://meme-suite.org/index.html (53). We downloaded the FASTQ data sets of RRBS for MCF7 cells from the ENCODE portal (54) (https://www.encodeproject.org/) with the following identifiers: ENCSR943EFS, and ENCSR939RXT; then avisualized with Integrative Genomics Viewer (IGV). Venn diagrams to identify the overlapping genes were generated using the Venny tool https://bioinfogp.cnb.csic.es/tools/venny/index.html. For RNA-Seq analysis, the RNA-Seq reads were normalized by RPKM and assembled by mapping reads directly to the annotated human reference genome using the DNASTAR SeqMan software (DNASTAR, Inc., Madison, WI). Differential gene expression levels were quantified using Fisher’s Exact Test Signal Search in the DNASTAR ArrayStar software package (DNASTAR, Inc., Madison, WI). Differentially expressed genes were filtered if they met the criterion for a two-fold change, a p-value that was less than.05 at a 95% confidence interval. For each comparison, genes were sorted based on fold change, from low to high. The results were ported into Excel spreadsheets where the log2 of the fold change for each gene was calculated.

### RNA Analysis *In Silico*

Relative RNA expression of 20 selected genes in breast cancer and normal adjacent tissue was downloaded from UCSC Xena platform on 11th of April 2020 (1,092 breast cancer primary tumors and 114 normal tissues).

### Liquid Chromatography/Mass Spectrometry (LC–MS/MS)

PC3 and MDA-MB-468 cells were cultured and seeded in p150 mm dishes at 37°C under atmospheric oxygen conditions. Once 70% confluent, cells were treated with DMSO, 2µM panobinostat, 10 µM Inhibitor-IV, 10 µM Inhibitor-VII, and 10 µM pracinostat for 45 min to 1.5 h and harvested in RIPA buffer (with complete protease inhibitor cocktail, 1 µM Trichostatin A and 1 mM nicotinamide). Protein concentration was quantified by the BCA method. Immunoprecipitation was performed using 4 μg of anti-MeCP2 antibody (Cell Signaling) and incubated for 2 h at 4°C. Protein A dynabeads (Invitrogen) were added to the immune-complex and incubated for 2 h at 4°C. IP protocol was followed as mentioned above. Beads were washed with RIPA buffer (four times) and autoclaved water (two times). Dry beads were shipped to Applied Biomics Inc. (Hayward, CA) for acetylation site identification by LC–MS/MS mass spectrometry on a fee-based service. The specific lysine residues that were acetylated, exhibited ion peaks at mass/charge (m/z) ratio of ~126 as summarized in **Figure 4A**.

### Statistical Analysis

Statistical analysis was performed using unpaired student’s t tests (Graph Pad Prism software) to assess whether differences observed in the various experiments were significant. All results are expressed as mean ± SEM and considered significant at *p <0.05, **p <0.01 and ***p <0.001.

## Results

### MeCP2 Binds Novel Genes in Breast Cancer Cells Associated With Diverse Biological Functions

**Figure 1:**
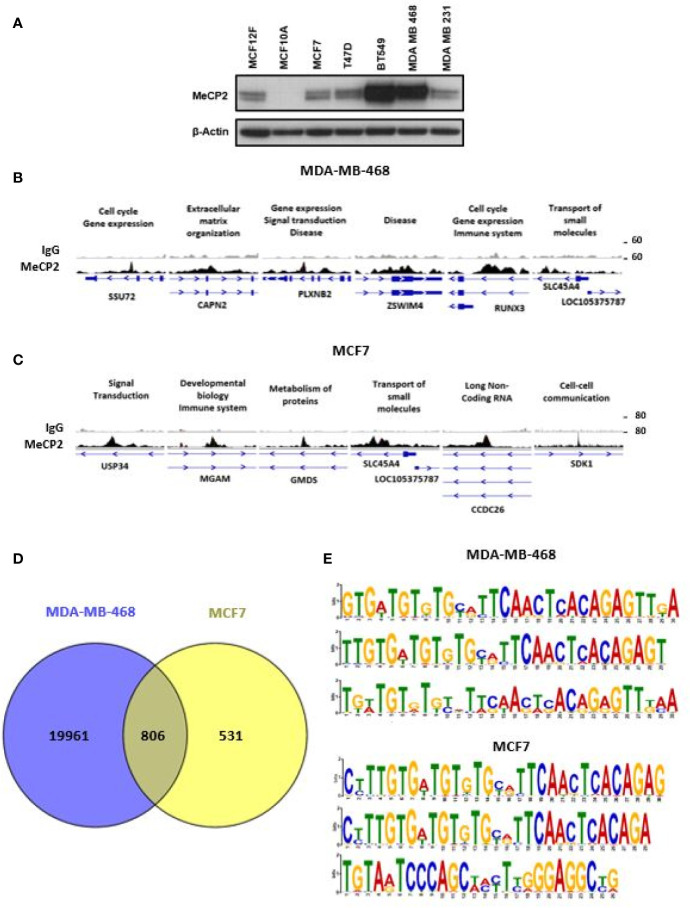
ChIP-Seq identified novel MeCP2 binding motifs. **(A)** MeCP2 expression in breast cancer cell panel including non-cancer cell lines MCF12F and MCF10A and breast cancer cell lines MCF7, T47D, BT 549, MDA-MB-468 and MDA-MB-231. **(B)** An assembly of IgG (first row) and MeCP2 (second row) ChIP-Seq data in MDA-MB-468 for the SSU72, CAPN2, PLXNB2, ZSWIM4, RUNX3, and SLC45A4 genes, visualized by IGV. **(C)** An assembly of IgG (first row) and MeCP2 (second row) ChIP-Seq data in MCF7 for the USP34, MGAM, GMDS, SLC45A4, CCDC26 and SDK1 genes, visualized by IGV. For **(B, C)** each column is 4,000 bp wide. The third rows show the gene nearest to the ChIP-Seq alignment including its location, and orientation. The medium thick dark lines are the UTRs of the gene and the thicker dark regions are exons followed by thin lines with arrows which are the introns. **(D)** Venn diagram representing overlap of MeCP2 ChIP-Seq peaks between MDA-MB-468 and MCF7 cells. **(E)** MEME-ChIP (Motif Analysis of Large Nucleotide Datasets) analysis of the MeCP2 binding sites identified MeCP2 specific motifs in MDA-MB-468 cells and MCF7 cells.

Since the discovery that MeCP2 regulates transcription and mutations in the gene cause Rett Syndrome, there has been considerable interest in what regulates its function and what downstream genes are targeted (55, 56). DNA methylation and its readers influence transcription activation and repression in a context-dependent manner depending on the genomic location of binding (57, 58). While this process is known to be frequently altered in cancers (59–61), many unknowns remain regarding the role of MeCP2 in regulating gene expression. Given abnormal DNA methylation in breast cancers (10, 12, 62, 63) and MeCP2 amplification in cancers (41), we wanted to identify new MeCP2 target genes and map novel sites of MeCP2 post-translational acetylation in breast cancer cells. We first examined MeCP2 protein expression in breast cancer cells and noted a range of expression across all lines with higher expression (64, 65) in MDA-MB-468 and BT-549 cells (**Figure 1A**). Both of these lines are derived from triple negative breast cancer (TNBC) which lack the expression of hormone receptors (ER and PR) and do not overexpress the growth factor receptor, HER2. To identify novel genomic targets of endogenous MeCP2, we performed MeCP2 ChIP-Seq analyses across two breast cancer cell lines (MCF7 and MDA-MB-468). These cells were chosen because they represent two different breast cancer subtypes and show relatively different MeCP2 protein expression levels. Also, inclusion of MCF7 in the ENCODE Project enabled comparison of our ChIP-Seq data with other publicly available data for epigenetic marks mapped in this cell line.

MeCP2 ChIP-Seq had not been done in MDA-MB-468 cells and our analysis revealed that MeCP2 binds to a wide spectrum of target genes (~20,000 in MDA-MB-468 and ~1,337 in MCF7 cells) ranging from miRNA, lncRNA, snRNA, processed and unprocessed pseudogenes, antisense and protein-coding genes. These genes are associated with a diverse range of cellular processes like gene expression, organization of the extracellular matrix, transport, or signal transduction, as shown in **Figures 1B, C**. In MeCP2 ChIP-Seq in MDA-MB-468 cells, we found that MeCP2 binds to multiple novel targets not previously associated with MeCP2 function in the context of breast cancer. Some of these included the following genes: a) SSU72 Homolog, RNA Polymerase II CTD Phosphatase (SSU72), a protein phosphatase that catalyzes the dephosphorylation of the C-terminal domain of RNA polymerase II (66); b) CAPN2 (Calpain 2), a calcium-sensitive cysteine protease (67); c) Plexin B2 (PLXNB2), a class B transmembrane receptor that participates in axon guidance and cell migration in response to semaphorins (68); d) Zinc Finger SWIM-Type Containing 4 (ZSWIM4); e) RUNX Family Transcription Factor 3 (RUNX3) a transcription factor that functions as a tumor suppressor and is frequently deleted or transcriptionally silenced in cancer (69, 70), and f) Solute Carrier Family 45 Member 4 (SLC45A4) (**Figure 1B**). Additionally, in MCF7 some of the notable genes included a) Ubiquitin Specific Peptidase 34 (USP34), a ubiquitin hydrolase that removes conjugated ubiquitin from AXIN1 and AXIN2, acting as a regulator of Wnt signaling pathway (71); b) Maltase–Glucoamylase (MGAM), an enzyme that plays a role in the digestion of starch (72); c) GDP-Mannose 4,6-Dehydratase (GMDS), an enzyme that participates in the synthesis of GDP-fucose from GDP-mannose (73); d) Solute Carrier Family 45 Member 4 (SLC45A4); e) CCDC26 Long Non-Coding RNA (CCDC26), a lncRNA class associated with Malignant Glioma and Astrocytoma (74, 75); and f) Sidekick Cell Adhesion Molecule 1 (SDK1) (**Figure 1C**). Moreover, 60% of the MCF7 loci (806 of 1,336) overlapped with MDA-MB-468 loci, including gene such as USP34, MGAM, GMDS, SLC45A4, CCDC26, and SDK1 (**Figure 1D**). We further analyzed the methylation status for genes in MCF7 cells for which publically available Reduced Representation Bisulfite Sequencing (RRBS) data was available (**Figure S1**). We found that MeCP2 binds to genes in MCF7 cells in regions where CpG methylation had been mapped such as SDK1, a cell adhesion molecule; Jagged 2 (JAG2), a Notch ligand; glycogenin 2 (GYG2), an enzyme involved in glycogen synthesis (**Figure 1C**, **Figure S1C**). These novel MeCP2 targets as well as others in **Figure S1** had not previously been linked with MeCP2, but have been linked with pathobiology associated with cancer (76–84) or genetic disorders such as Leigh syndrome (85) and Raine syndrome (86, 87). We also found that MeCP2 binds to genomic regions devoid of CpG methylation such as for USP34, MGAM, GMDS, SLC45A4, SSU72, CAPN2 and PLXN2 (**Figures S1B–C**). Similarly, while these are novel targets of MeCP2, many have been implicated in diverse cancers (67, 71, 88–92). This further shows the complexity of MeCP2 binding across the genome. To identify the DNA motifs associated with MeCP2 genomic binding, we analyzed the genomic fragments sequenced in our MeCP2 ChIP-Seq analyses performed in triplicate. The MEME-ChIP analysis revealed a motif consistent across three independent experiments for both MDA-MB-468 and MCF7 cells (**Figure 1E** and **Table 3**).

**Table 3:** MeCP2 binding motifs.

	CONSENSUS	Width	Fragments	E-value
**MDA-MB-468**				
Replicate #1	GTGATGTGTGYRTTCAACTCACAGAGTTGA	30	3232	8.1e-3515
Replicate #1	CTAGACAGAAKMATTCTCAGAAACTT	26	3568	1.1e-2663
Replicate #1	TTWCAYAGAGCAGWTTTGAAACACTCTTT	29	2923	6.5e-2645
				
Replicate #2	TTGTGATGTGTGYRTTCAACTCACAGAGT	29	3102	1.4e-3473
Replicate #2	AARCTAGACAGAAKVATTCTCAGAAA	26	3210	1.3e-2530
Replicate #2	AACVTTYCTTTTCAYAGAGCAGWTTKGAAA	30	2685	5.0e-2508
				
Replicate #3	TGWTGTGTGYDTTCAACTCACAGAGTTKAA	30	1762	9.7e-1782
Replicate #3	TAGACAGAAKMATTCTCAGWAACTTCYTTG	30	1325	5.0e-1682
Replicate #3	TYCTTTWCAYAGAGCAGWTTKGAAACACTC	30	1085	3.3e-1262
**MCF7**				
Replicate #1	CTTTGTGATGTGTGYRTTCAACTCACAGA	30	815	7.2e-2719
Replicate #1	YTAGACAGARBARTTCTSARAMACTY	26	1530	2.0e-2663
Replicate #1	GCAAGTGAKATTTVRACCKCTTTGAGGYC	30	658	4.7e-1616
				
Replicate #2	CTTTGTGATGTGTGYRTTCAACTCACAGA	29	698	5.1e-2488
Replicate #2	YTAGACAGARBARTTCTSARAMAC	24	1273	3.1e-2310
Replicate #2	GMWTKGARKSSAATGGWRKRR	21	1428	7.5e-1387
				
Replicate #3	TGTAATCCCAGCWMYTYGGGAGGCYG	26	6885	2.8e-1157
Replicate #3	TTTTTKTWTTTTTWKTWGAGAC	22	7314	1.3e-709
Replicate #3	GCCACCTCGCCCGGC	15	7915	1.6e-485

### MeCP2 Localizes to Novel Genes and Regulates Their Expression

**Figure 2:**
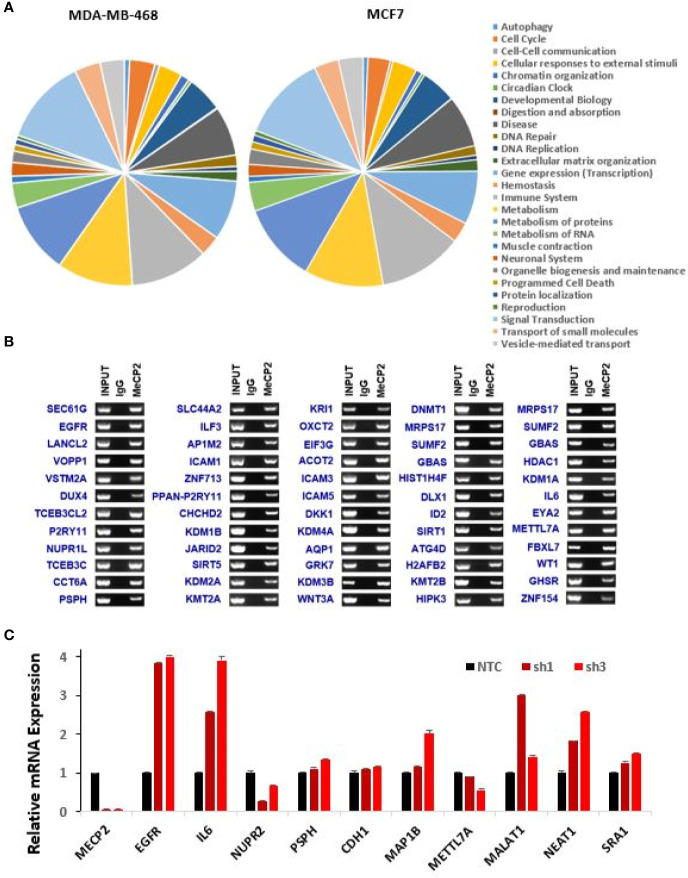
Novel genes targeted and regulated by MeCP2 in breast cancer. **(A)** Pie graph showing enriched pathways of ChIP-hits for MDA-MB-468 and MCF7 cell lines generated by Reactome pathway analysis. **(B)** ChIP-Seq experiments for IgG and MeCP2 were performed in MDA-MB-468. ChIP followed by end-point PCR was performed to validate 60 genes targeted by MeCP2. **(C)** Representative of two-independent RT-qPCR-based analysis to evaluate expression changes of MeCP2, EGFR, IL6, NUPR2, PSPH, CDH1, MAP1B, METTL7A, MALAT1, NEAT1, AND SRA1 genes in MDA-MB-468 (NTC, sh1 MeCP2, and sh3 MeCP2) cells. Transcript levels were normalized to actin transcript levels.

We further determined the global occupancy of MeCP2 with respect to cellular functions and performed pathway analysis to identify the core pathways associated with the newly identified target genes in MDA-MB-468 and MCF7. We observed an enrichment of the gene expression, immune system, metabolism, metabolism of proteins, and signal transduction pathways (**Figure 2A**). We further randomly chose more than 100 genes identified in the triplicate analysis of MeCP2 ChIPseq in MDA-MB-468 cells and validated MeCP2 binding *via* MeCP2 ChIP-PCR, some of which are shown in **Figure 2B**. Consistent with our MeCP2 ChIPseq analyses, we found *via* MeCP2 ChIP-PCR that MeCP2 localizes to various gene promoters involved in diverse biological processes such as immune system regulation (IL6, ICAM3, and ICAM5), signal transduction (EGFR, WNT3A, and DKK1), transcription (KMT2A, SIRT1, HDAC1, DNMT1), developmental biology (DUX4) and lncRNAs (MALAT-1 and NEAT1) in MDA-MB-468 cells (**Figure 2B**). Several of the lncRNA targets identified are poorly studied, so we examined transcript expression patterns of some associated with MeCP2 MALAT1 and NEAT1 and established expression patterns across a panel of breast cancer cells. To determine whether MeCP2 depletion would lead to a change in expression of novel gene targets, MeCP2 was stably depleted with two different shRNA (sh1 and sh3) in MDA-MB-468 cells (**Figure S2A**). We also observed by quantitative RT-qPCR a change in mRNA expression of novel targets in which were validated for knockdown (**Figure S2A**). A minimum of three independent experiments showed that depletion of MeCP2 caused a change in the expression of several of the genes whose promoter it bound. We found that knockdown of MeCP2 in MDA-MB-468 cells caused an increase in some genes such as *IL6*, *KDM3B*, *HIPK3*, *KDM3A*, *EGFR*, and *KMT2B* and a reduction others such *NUPR1L* (also known as *NUPR2*), *METTL7A*, *PSPH*, *LANCL2*, *MRPS17* and HDAC1. (**Figure 2C** and **Figure S2B**). Together these results show the complexity of MeCP2-mediated regulation of gene expression.

### MeCP2 Targets Genes With Differential Expression Between Breast Cancer and Normal Samples

**Figure 3:**
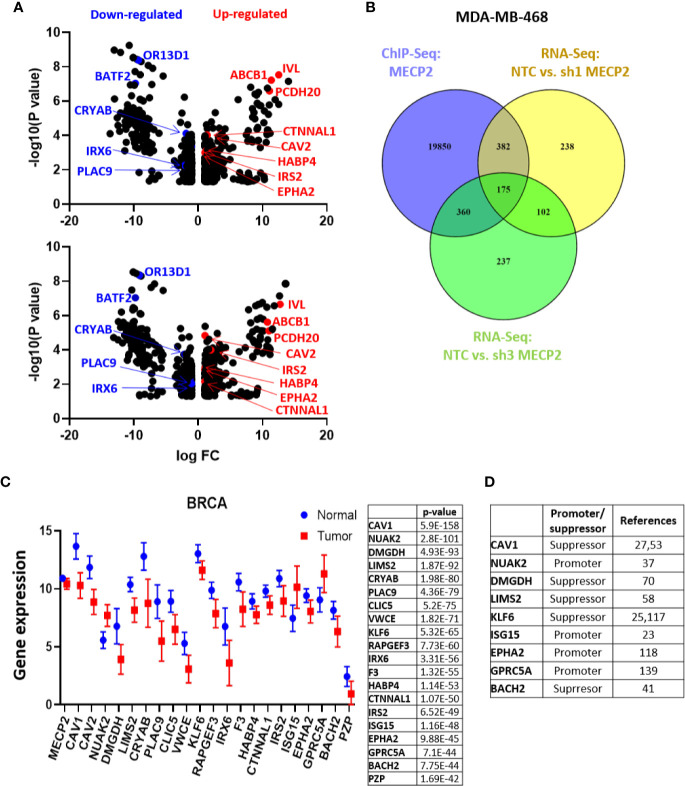
MeCP2 depletion induces transcriptional changes in MDA-MB-468 cells. **(A)** A volcano plot showing up-regulated (red) and down-regulated genes (blue) in RNA-Seq analysis between NTC and sh1 MeCP2 (upper panel) and NTC vs sh3 MeCP2 (bottom panel). **(B)** Venn diagram representing overlap of MeCP2 ChIP-Seq peaks and RNA-Seq analysis in MDA-MB-468 cells. **(C)** Differential expression of 20 selected genes of the 175 genes common in MDA-MB-468 ChIP-Seq and RNA-Seq. **(D)** Role of selected genes in cancer tumorigenesis.

To evaluate the global effect of MeCP2 on RNA expression we performed RNA-Seq in MDA-MB-468 cells. We analyzed three independent experiments of non-targeting control (NTC) versus sh1 MeCP2 and NTC versus sh3 MeCP2 and found changes in 899 genes and 875 genes, respectively (**Figure 3A**). Overlap of ChIP-Seq hits and RNA-Seq hits showed 175 potential transcriptional targets of MeCP2 (**Figure 3B**). A pathway enrichment analysis of these potential targets showed their participation in the immune system, metabolism, metabolism of proteins, and signal transduction, among other pathways (**Table 4**). Moreover, these genes were differentially expressed in normal vs. breast cancer tissue (**Figure 3C**), and several of these target genes have been previously reported to be tumor suppressors (93–99) while others were reported to be oncogenes (100–103) (**Figure 3D**).

**Table 4:** Pathway analysis of the intersecting genes of MeCP2 ChIP-Seq and RNA-Seq.

Pathway identifier	Pathway name	Genes
R-HSA-9612973	Autophagy	RRAGD
R-HSA-1640170	Cell Cycle	DMGDH;MECP2;DACH1;PIM1;L1CAM;PHLDA1
R-HSA-8953897	Cellular responses to external stimuli	DMGDH;DACH1;NCF1;RRAGD;ADRA1B;CRYAB
R-HSA-1266738	Developmental Biology	DMGDH;NRP2;ADGRV1;PLXND1;SEMA3E;IRS2;L1CAM;MECP2;DACH1;COL4A1;ITGA10;COL5A2;PI3;KRTAP3-1;EPHA2
R-HSA-1643685	Disease	JAG2;DMGDH;PDGFRA;FLII;PCDH8;IRS2;ISG15;FMO3;L1CAM;MECP2;NT5E;DACH1;TBXAS1;PIM1;GPC2;LRAT;GGT1;TMEM45A;MUC6
R-HSA-73894	DNA Repair	TNNT2;ISG15
R-HSA-1474244	Extracellular matrix organization	MECP2;MMP25;COL4A1;ITGB4;COL7A1;ITGA10;COL5A2;P3H2;LTBP1
R-HSA-74160	Gene expression (Transcription)	DMGDH;MECP2;GRIN2A;DACH1;CAV1;IGFBP3;RRAGD;PCDH8;NALCN;ANKRD6
R-HSA-109582	Hemostasis	PDGFRA;CAV1;TFPI2;L1CAM;F3;PSG9;CEACAM3;PSG8;ISLR;PSG5;PSG4;ITGA10;HABP4;TIMP3;SLC16A8;KIF1A;RAPGEF3;CD177
R-HSA-168256	Immune System	DMGDH;NCF1;MAST4;ITGB4;FBXO27;IRS2;CST6;MECP2;C4B;MMP25;DACH1;PIM1;SNCG;PI3;MUC6;CD177;PDGFRA;OSBPL6;IL34;LIF;ISG15;OLFM4;CEACAM3;BST2;RAPGEF3
R-HSA-1430728	Metabolism	ARL9;DMGDH;CLIC5;NRP2;MOGAT2;WNK4;LTBP1;NALCN;NT5E;DACH1;GPC2;LRAT;ST3GAL6;SLC16A8;GGT1;UGT1A7;GGT2;OSBPL6;CAV1;CYP4B1;FMO3;KMO;TIAM2;AKR1B10;TBXAS1;RAPGEF3
R-HSA-392499	Metabolism of proteins	DMGDH;OSBPL6;NCF1;FBXO27;IGFBP3;PCDH8;P3H2;LTBP1;NALCN;ZDBF2;MECP2;NT5E;LGALS1;DACH1;COL4A1;COL7A1;TNNT2;ATP13A4;CPE;ST3GAL6;FOLR1;TMEM45A;MUC6;RABL6
R-HSA-1852241	Organelle biogenesis and maintenance	RABL6
R-HSA-9609507	Protein localization	P3H2
R-HSA-162582	Signal Transduction	DMGDH;CLIC5;NRP2;NCF1;PLXND1;FLII;STK39;IRS2;GRIK2;ADRA1B;NALCN;MECP2;NT5E;DACH1;GPC2;LRAT;ST3GAL6;NTSR1;JAG2;PDGFRA;EDN1;CAV2;PDE6G;CAV1;GPR55;L1CAM;F3;TIAM2;FOSL1;AKR1B10;COL4A1;COL5A2;PPP1R1B;RRAGD;RAPGEF3;RABL6
R-HSA-382551	Transport of small molecules	DMGDH;SLC15A1;DACH1;TTYH1;ATP10D;WNK4;ATP13A4;ATP13A5;SLC16A8;SLC12A7;NALCN
R-HSA-5653656	Vesicle-mediated transport	TBC1D2;MAST4;COL4A1;COL7A1;P3H2;HSF4;KIF1A;FOLR1;KIAA0319;RABL6

### Endogenous MeCP2 Is Acetylated at Key Lysine Residues and KDI Further Influence Its Acetylation Patterns

**Figure 4:**
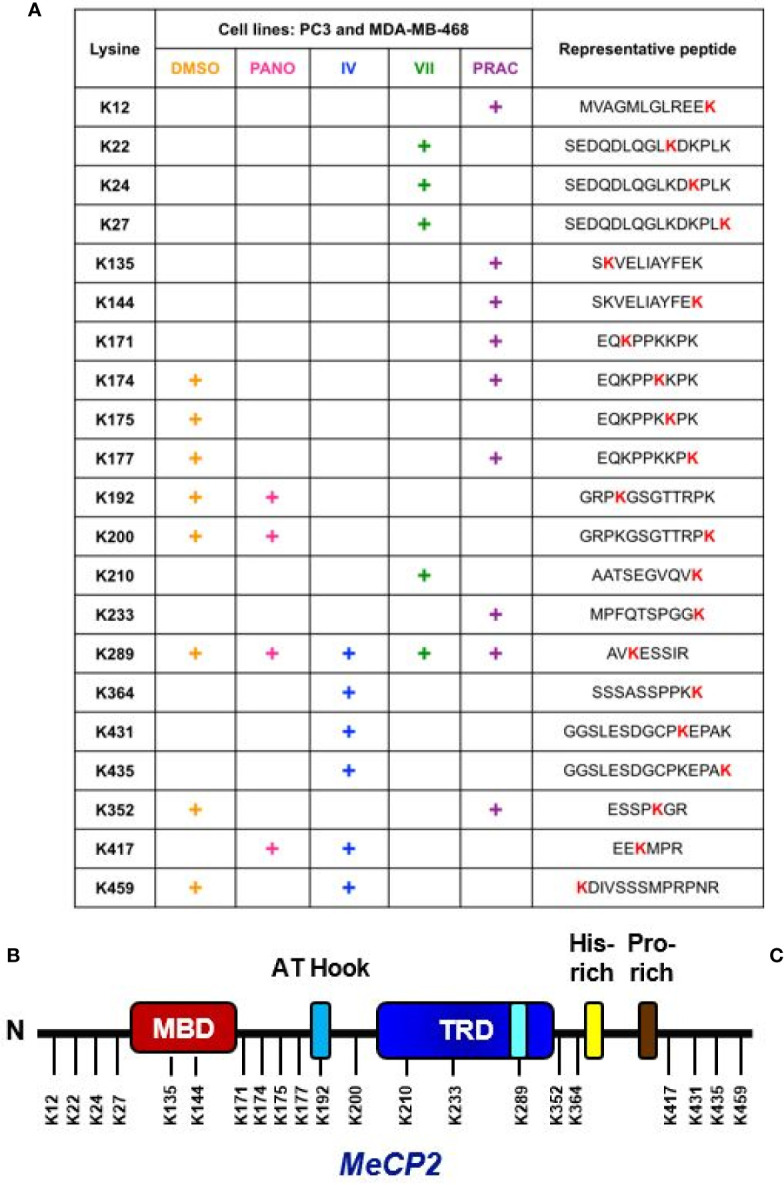
Endogenous MeCP2 is acetylated at key lysine residues. **(A)** The table indicates putative lysine residues that were found to be acetylated on MeCP2 under basal condition (DMSO) and upon deacetylase inhibition using 2 µM panobinostat (PANO), 10 µM SIRT1/2 Inhibitor-IV (IV), 10 µM SIRT1/2 Inhibitor-VII (VII), and 10 µM pracinostat (PRAC) and showed ion peaks at mass/charge (m/z) ratio of ∼126 in PC3 and MDA-MB-468 cells. **(B)** Approximate representation of the position of acetylated lysine (K) residues on MeCP2 conserved domain is shown. N, N-terminal; MBD, Methyl-binding-domain: A-T Hook domain: TRD, Transcriptional repression domain: His-rich, Histidine-rich domain: Pro-rich, Proline-rich domain, C, C-terminal.

We previously reported that MeCP2 undergoes acetylation on Lys-171 in both MCF7 and RKO cells. We further demonstrated that a K171 acetylation mimetic did not perturb binding to select gene targets, but it diminished interaction of MeCP2 with binding partners such as ATRX and HDAC1 in colorectal cancer cells (44). *In vivo* and *in vitro* studies have demonstrated the importance of MeCP2 post-translational regulation (45, 104–107), yet little has been done to comprehensively map novel MeCP2 PTMs. In the current study we wanted to extend our analyses and provide a comprehensive map of post-translational acetylation in other cancer cell line models. In order to further understand how MeCP2 is post-translationally regulated in TNBC breast and prostate cancer cell lines, we systematically identified the specific lysines on endogenous MeCP2 where acetylation was induced upon lysine deacetylase inhibition (KDACi). We inhibited SIRT1, a class III lysine deacetylase, using 10 µM Inhibitor-IV or 10 µM Inhibitor-VII, as well as the class I/II/IV lysine deacetylases using 2 µM panobinostat and 10 µM pracinostat. Given the links between DNA methylation and/or aberrant expression of DNA methylation readers in prostate cancer (4, 8) and TNBC (12, 42), we focused on two model lines representing each cancer, PC3 and MDA-MB-468, respectively. Next, we performed immunoprecipitation of endogenous MeCP2 and analyzed the samples using LC–MS/MS. **Figure 4A** summarizes the specific lysine residues that were acetylated and exhibited ion peaks at mass/charge (m/z) ratio of ~126 under basal (vehicle control) and KDI-induced conditions (i.e., cells treated with panobinostat, Inhibitor-IV, Inhibitor-VII, and pracinostat) (also see **Figure S3**). The mass spectrometry analyses showed that endogenous MeCP2 was acetylated at eight lysine residues under basal conditions (i.e., vehicle control) with induction in acetylation on K417 with 2 µM panobinostat; K364, K417, K431, K435 with 10 µM Inhibitor-IV; K22, K24, K27, K210 with 10 µM Inhibitor-VII; and K12, K135, K144, K171, K233 with 10 µM pracinostat. We found changes in acetylation patterns induced by exposure to both pre-clinical KDIs such as SIRT1 inhibitors and pracinostat as well as an FDA-approved inhibitor, panobinostat, which is used in the clinic to treat leukemias and lymphomas (23, 108). Interestingly, some of the lysine residues detected as acetylation sites (K22 and K135) were also sites mutated in Rett Syndrome. Moreover, some of the lysine residues detected as acetylation sites (such as K135), have been previously reported as sites linked with ubiquitination (4). We found acetylated lysine residues across the length of the protein, including at the N-terminus, in the methyl-binding domain (MBD), in the intermediate domain (ID) and the transcriptional repression domain (TRD) as well at the C-terminus region (**Figure 4B**). Together, these results indicate that MeCP2 is acetylated under basal and KDI-induced conditions in multiple cancer cell lines.

**Figure 5:**
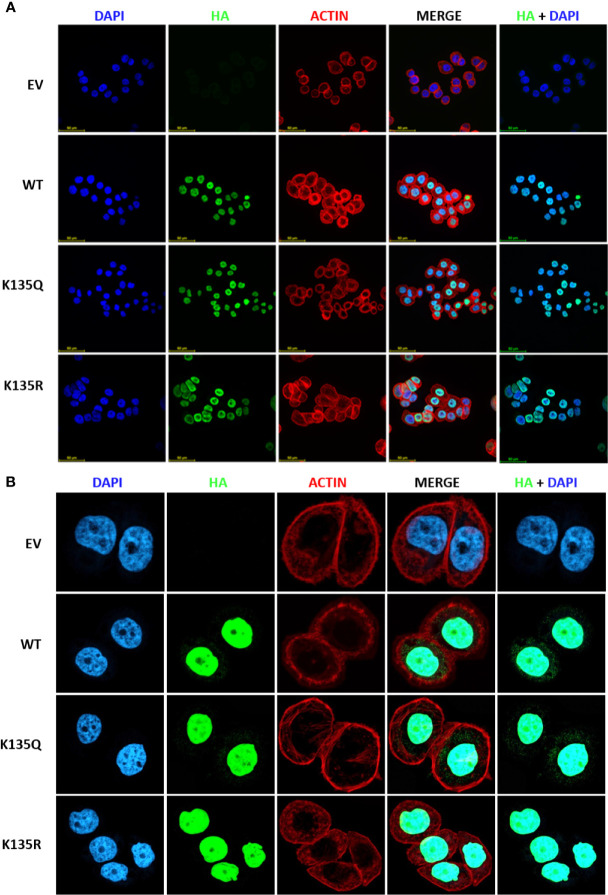
Acetylation on K135 lysine residue does not alter sub-cellular localization of MeCP2 but influences its binding at gene promoters in MDA-MB-468 cells. **(A)** Immunofluorescence staining of empty vector (EV), HA-tagged MeCP2 (WT), K135R mutant (HA-K135R), and K135Q mutant (HA-K135Q) cells. Merge of HA-MeCP2 (green) and nuclear staining (blue) proteins is shown as HA/DAPI for each of the cells. Merge of DAPI (blue), HA-MeCP2 (green), and actin (red) is shown as MERGE for each of the cells. **(B)** Immunofluorescence staining of EV, WT, HA-K135R, and K135Q cells at higher magnification.

Next, we wanted to determine the impact of K135 acetylation on MeCP2 subcellular localization. We chose to study this site since it is situated in a highly conserved MBD domain and is a residue mutated in Rett syndrome patients. In order to probe the functional significance of MeCP2 acetylation, we generated HA-tagged wild-type MeCP2, HA-tagged deacetylation mimetics (K135R), HA-tagged acetylation mimetics (K135Q). Once the mutations were confirmed by sequencing, we then transfected and selected MDA-MB-468 cells with the plasmids for stable expression. Overexpression of HA-tagged MeCP2 constructs was confirmed by protein expression of WT and point mutants in MDA-MB-468 cells (**Figure S4B**). Using immunofluorescence assays, we detected that HA-tagged wild-type MeCP2, deacetylation mutants (K135R), and acetylation mutants (K135Q) were mostly in the nucleus of stably expressing MDA-MB-468 cells (**Figure 5**). These data demonstrate that post-translational acetylation on K135 lysine residue does not alter MeCP2 sub-cellular localization and calls for future studies to examine the role of acetylation at this residue as well as others identified in this report.

## Discussion

The present study provides valuable insight on two important fronts. First, we identify novel genes that are subject to MeCP2-mediated regulation. Second, we provide a comprehensive identification of novel sites of post-translational acetylation associated with different cancer types and in response to multiple classes of deacetylase inhibitors. Concerning genomic analyses, these findings are important because we identify novel MeCP2 target genes linked with tumor progression which were not previously linked with MeCP2. While global DNA hypomethylation frequently occurs during tumorigenesis (60, 61), the promoters of TSGs may undergo hypermethylation (109–111) and these aberrant changes in both the marks and the enzymes that modify them are being intensively examined for novel therapies (112–116). These epigenomic changes may instigate genomic instability or generate a heritable molecular signature, which enables tumor progression, so identification of novel genomic targets of MeCP2 is very important (117–119).

Previous reports linked MeCP2 expression with ER status (3) and with BRCA1 promoter silencing (120), which provided further rationale for assessing genome-wide MeCP2 profiling in both MCF7 and MDA-MB-468 cells, which represent two subtypes of breast cancer. We found that MeCP2 binds to multiple regions of genes, including promoters, exons, and introns. These novel targets have been associated with a wide range of regulatory and signaling pathways. We found that there was an overlap of around 800 genes between the two cell lines, and there were distinct MeCP2 binding motif enrichments between both cell lines. We observed that not only did MeCP2 bind many novel gene targets, but its depletion also led to both increases and decreases in their corresponding RNA transcripts. This is especially important given that studies demonstrate MeCP2 binds to methylated cytosines and hydoxymethylated cytosines in mCH dinucleotides, a property wherein many unknowns remain (31–33, 35, 36, 38, 121). We discovered that MeCP2 localizes at various gene promoters involved in diverse processes such as autophagy (ATG4D), immune cell regulation (IL6), chromatin organization (KDM3B, KDM2A, KMT2B, KMT2A, KDM1A, HDAC1, HIST1H4F), circadian clock (SIRT1), developmental biology (EGFR, DKK1, SUMF2), extracellular matrix organization (ICAM5, ICAM3, ICAM1) and metabolism (EIF3G, SLC44A2, SUMF2, OXCT2, ACOT2, PSPH).

Recently, MeCP2 was shown to be amplified in human tumors and can mimic the function of activated Ras in cancer models (42), and also acts as a critical bridge linking information encoded in methylated DNA to epigenetic regulators (40, 122). Although MeCP2 binds methyltransferases (25), co-repressors (123) histone deacetylases (26), chromatin modulators (41), and long noncoding RNAs (lncRNAs) (124, 125) and other epigenetic regulators (4), much remains unknown about what regulates these interactions and what regulates binding to mCG vs. mCA dinucleotides as well as methylation-independent binding (126). However, our previous report provided some of the first insight into the role of post-translational regulation of MeCP2 binding to co-repressor proteins. We found that K171 acetylation regulates MeCP2 interaction with HDAC1 and ATRX (44). It is worth noting that the severity of Rett syndrome is influenced by the location of the mutation. For example, the site of the mutation can strongly impact cognitive and psychomotor skills as well as neonatal encephalopathy and death. Some mutations have been shown to disrupt conserved AT-hook regions and cause differential localization of α-Thalassemia/Mental Retardation Syndrome X-Linked (ATRX) (23). ATRX is a critical SWI/SNF-like chromatin remodeler (127) and our previous report demonstrates that a novel PTM on MeCP2 regulates MeCP2-ATRX binding, which is a critical aspect of MeCP2 function (43). Our present study identified an additional 17 novel sites of post-translational MeCP2 acetylation in triple-negative breast cancer and prostate cancer cell lines. Notably, nine of these lysines (K12, K135, K144, K177, K210, K233, K289, K364, K352, and K417) have been shown to be mutated in patients with Rett Syndrome. We identified four of these sites in our previous study mapping MeCP2 acetylation in MCF7 breast cancer cells and RKO colon cancer cells (K22, K135, K171, and K289) (43). Based on previous findings one may reason that one or more of these novel PTMs may be influencing MeCP2 function in cancer progression. Another example of the impact of post-translational regulation comes from transgenic models involving single MeCP2 serine residues that undergo post-translational regulation which show distinct neurological defects (106, 128), and phosphorylation of specific serine residues is enriched at specific gene promoters (104). However, much less is known about the role of MeCP2 acetylation as a regulatory switch in any context. Our more thorough mapping of novel MeCP2 acetylation PTMs performed here is a first step in defining their functional significance which is beyond the scope of the present study. Based on MeCP2 acetylation patterns induced by the various pre-clinical or clinical lysine deacetylase inhibitors, it is likely that KDACi’s that target class I/II vs. class III HDACs will influence MeCP2 function in both common and distinct ways. Based on acetylation mapping one can also reason that MeCP2 interaction with different KDACs may lead to important role in cell-type-specific biology driven by unique acetylation patterns. We previously demonstrated that lysine acetylation serves as a regulatory switch in Wnt pathway signaling (129, 130) and cancer-associated steroidogenesis (131, 132). The current study provides yet another example of the scope of post-translational acetylation and may help explain how SIRT1 preferentially targets active (133, 134) vs. repressed genes (135) depending on its deacetylation of specific non-histone partners (136, 137). Future work may identify more factors involved in this SIRT1-MeCP2 regulatory network, and through such work, our understanding of the key molecular relationships in cancer may lead to deeper understanding of the mechanism of action of epigenetic therapies and KDAC inhibitors.

## Data Availability Statement

Sequences and processed ChIP-Seq and RNA-Seq data files were deposited in the NCBI Gene Expression Omnibus (GEO) database under accession number GSE160150 and the BioProject: PRJNA667107.

## Author Contributions

IC-P, DV, MS, SP, LC, DM, MF, and KP discussed and designed the experiments. IC-P, DV, MS, SP, LC, DM, and MF performed experiments and/or analyzed data. IC-P, MS, DM, and KP wrote and edited the paper with input from all authors. IC-P, MS, SP, LC, DM, MF, JN, SA, RL, FR, and KP reviewed and revised the paper. All authors contributed to the article and approved the submitted version.

## Funding

This work was supported by the National Institute of Health (CA155223) to KP and a Cancer Prevention and Research Institute of Texas (CPRIT), Recruitment of Rising Stars Award (RR410008) to KP.

## Conflict of Interest

The authors declare that the research was conducted in the absence of any commercial or financial relationships that could be construed as a potential conflict of interest.

## Supplementary Material

Data Sheet 1 MeCP2 acetylation site identification by LC-MS/MS mass spectrometry in MDA-MB-468 cells.

Data Sheet 2 MeCP2 acetylation site identification by LC-MS/MS mass spectrometry in PC3 cells.

Supplementary Figure 1 MeCP2 binds to unmethylated DNA **(A)** An assembly of IgG (first row), MeCP2 (second row) ChIP-Seq, and RRBS (third row) data in MCF7 for USP34, MGAM, GMDS, SLC45A4, CCDC26 and SDK1 genes, visualized by IGV. Each column is 4,000 bp wide. The third rows show the gene nearest to the ChIP-Seq alignment including its location, and orientation. The medium thick dark lines are the UTRs of the gene and the thicker dark regions are exons followed by thin lines with arrows which are the introns. **(B)** Schematic representations of the top ChIP-Seq hits where the red box indicates the MeCp2 binding, the white circles indicated methylated regions identified by RRBS and the medium thick blue lines are the UTRs of the gene and the thicker blue regions are exons followed by thin lines which are the introns. **(C)** Table of MeCp2 ChIP-Seq hits summarizing the presence/absence of CG methylation identified by RRBS in MCF7 cells.

Supplementary Figure 2 Validation of MeCP2 knockdown. **(A)** Left panel. Western blot to evaluate the protein levels of MeCP2 in MDA-MB-468 (NTC, sh1 MeCP2, and sh3 MeCP2) cells. Right panel. RT-qPCR analysis to evaluate expression of MeCP2 in MDA-MB-468 (NTC, sh1 MeCP2, and sh3 MeCP2) cells. Transcript levels were normalized to actin transcript levels. **(B)** Representative of two-independent RT-qPCR-based analysis to evaluate expression changes of NUPR2, PSPH, LANCL2, MRPS17, HDAC1, KDM3B, HIPK3, KDM3A, EGFR and KMT2B genes in MDA-MB-468 (NTC and sh MeCP2) cells. Transcript levels were normalized to actin transcript levels.

Supplementary Figure 3 Pharmacological inhibition of lysine deacetylases and key lysine residues acetylated on endogenous MeCP2. Acetylation of MeCP2 detected by Western blotting. **(A)** PC3 cells were treated with deacetylase inhibitors: DMSO as vehicle control, and SIRT1/2 Inhibitor-IV for a short time period range from 10 min to 1:15 h. **(B)** MDA-MB-468 were treated with deacetylase inhibitors: DMSO as vehicle control, and SIRT1/2 Inhibitor-IV for a short time period range from 10 to 120 min. **(C)** MDA-MB-468 were treated with deacetylase inhibitors: DMSO as vehicle control, and with various doses of SIRT1/2 Inhibitor-IV. For all immunoprecipitations equal amount of protein were loaded for each immunoprecipitation set up using acetyl-lysine (Ac-K) antibody as per protocol. Acetylation of MeCP2 was detected by Western blotting along with positive control, whole cell extract (WCE) using MeCP2 specific antibody. Species-matched IgG was used as a negative control. IgG heavy chain (IgG Hc) was blotted for as a control for equal antibody loading for immunoprecipitation and GAPDH for WCE. **(D)** The table indicates putative lysine residues that were found to be acetylated on MeCP2 under basal condition (DMSO) and upon deacetylase inhibition using 2 µM panobinostat (PANO), 10 µM Inhibitor-IV (IV), 10 µM Inhibitor-VII (VII), and 10 µM pracinostat (PRAC) and showed ion peaks at mass/charge (m/z) ratio of ∼126 in PC3 and MDA-MB-468 cells.

Supplementary Figure 4 Expression profile of lncRNA across normal and breast cancer cell lines. **(A)** RNA samples were extracted and converted to cDNA by reverse transcriptase enzyme. RT-PCR was performed to determine the expression of MALAT-1, MEG3, NEAT-1, CDKN2B, GAS5, SRA1, MIR31HG LncRNAs, and Beta actin as positive control in MCF12F normal breast cells and MCF7, BT549, MDA-MB-468, MDA-MB-231 and T47D breast cancer cell lines. **(B)** Stable expression of empty vector (EV), HA-epitope tagged MeCP2 wild type (WT), HA-tagged deacetylation mutants (K to R), HA-tagged acetylation mutants (K to Q) on K135 lysine residues in MDA-MB-468 cells.

## Abbreviations

ChIP, Chromatin immunoprecipitation; ChIP-Seq, Chromatin Immunoprecipitation Sequencing; ENCODE, Encyclopedia of DNA Elements; FDA, U.S. Food and Drug Administration; HDAC, histone deacetylase; HMT, histone methyltransferase; KDI and KDACi, lysine deacetylase inhibitors; lncRNA, long non-coding RNA; MBD, methyl-CpG binding domain; MeCP2, methyl-CpG-binding protein 2; NTC, non-targeting control; PDX, Patient-Derived Xenograft; PTM, post-translational modifications; RNA-Seq, RNA-Sequencing; sh RNA, short hairpin RNA; SIRT1, Sirtuin 1; TNBC, triple negative breast cancer; TRD, transcriptional repression domain.
