# Supplementary figures and images for: Identification of Novel MeCP2 Cancer-Associated Target Genes and Post-Translational Modifications
Source: Front Oncol. 2020 Dec 10;10:576362. doi: 10.3389/fonc.2020.576362 (PMC7758440; doi:10.3389/fonc.2020.576362)

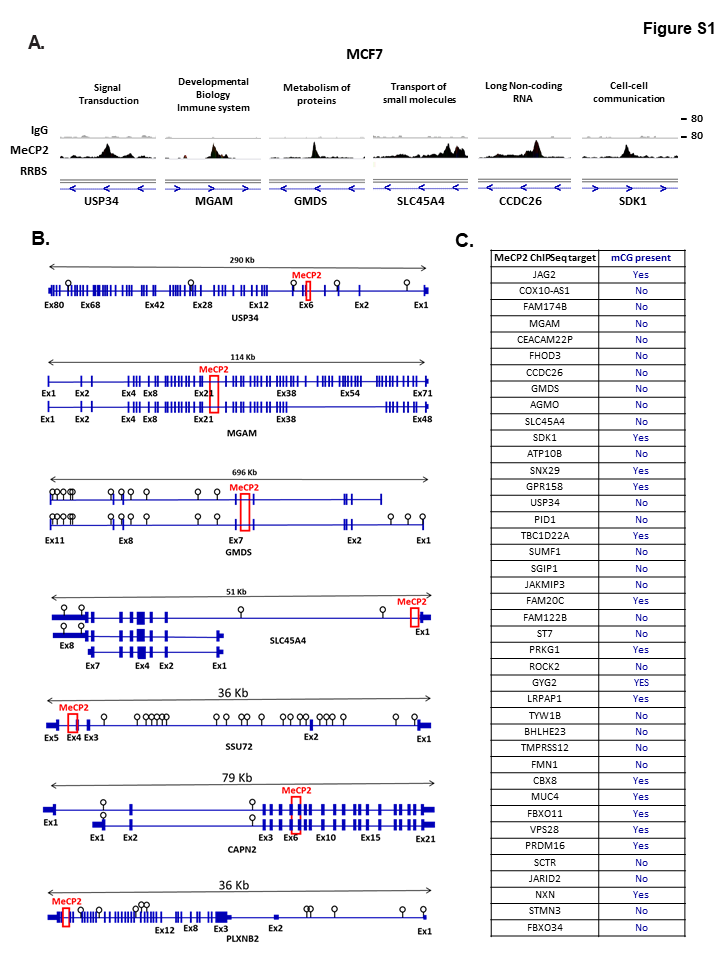

Supplement: Supplementary Figure 1 — MeCP2 binds to unmethylated DNA (A) An assembly of IgG (first row), MeCP2 (second row) ChIP-Seq, and RRBS (third row) data in MCF7 for USP34, MGAM, GMDS, SLC45A4, CCDC26 and SDK1 genes, visualized by IGV. Each column is 4,000 bp wide. The third rows show the gene nearest to the ChIP-Seq alignment including its location, and orientation. The medium thick dark lines are the UTRs of the gene and the thicker dark regions are exons followed by thin lines with arrows which are the introns. (B) Schematic representations of the top ChIP-Seq hits where the red box indicates the MeCp2 binding, the white circles indicated methylated regions identified by RRBS and the medium thick blue lines are the UTRs of the gene and the thicker blue regions are exons followed by thin lines which are the introns. (C) Table of MeCp2 ChIP-Seq hits summarizing the presence/absence of CG methylation identified by RRBS in MCF7 cells. [file Image_1.tif]

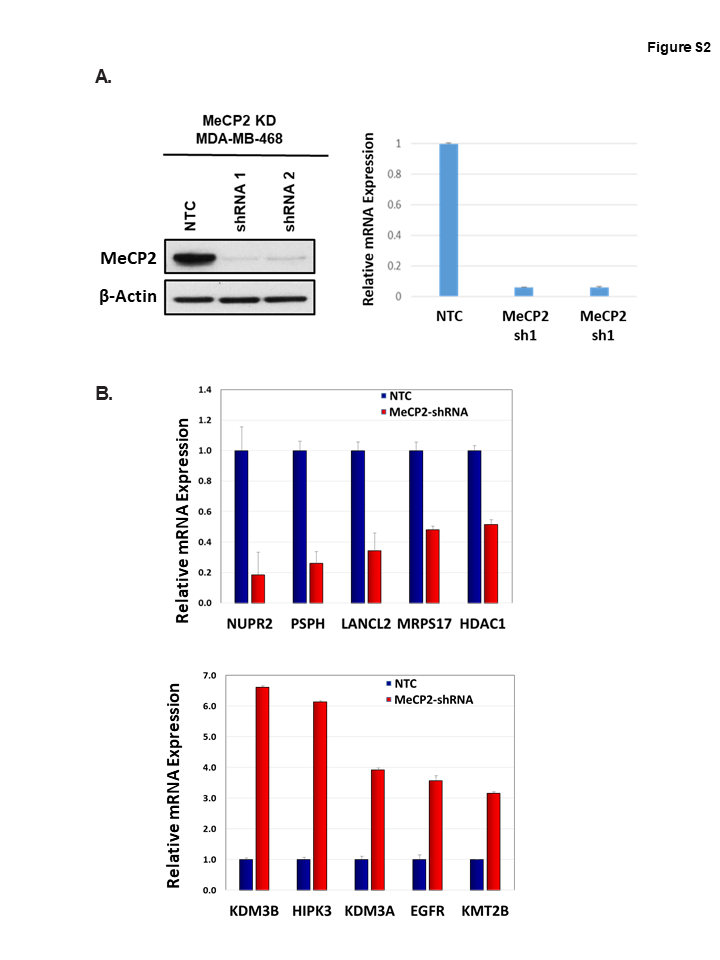

Supplement: Supplementary Figure 2 — Validation of MeCP2 knockdown. (A) Left panel. Western blot to evaluate the protein levels of MeCP2 in MDA-MB-468 (NTC, sh1 MeCP2, and sh3 MeCP2) cells. Right panel. RT-qPCR analysis to evaluate expression of MeCP2 in MDA-MB-468 (NTC, sh1 MeCP2, and sh3 MeCP2) cells. Transcript levels were normalized to actin transcript levels. (B) Representative of two-independent RT-qPCR-based analysis to evaluate expression changes of NUPR2, PSPH, LANCL2, MRPS17, HDAC1, KDM3B, HIPK3, KDM3A, EGFR and KMT2B genes in MDA-MB-468 (NTC and sh MeCP2) cells. Transcript levels were normalized to actin transcript levels. [file Image_2.tif]

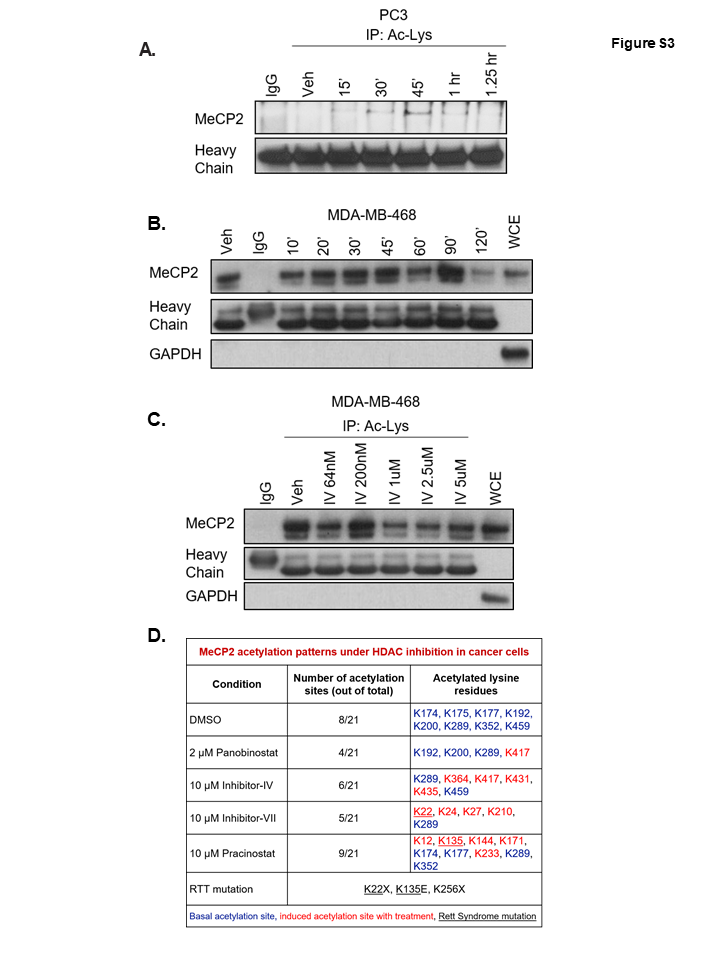

Supplement: Supplementary Figure 3 — Pharmacological inhibition of lysine deacetylases and key lysine residues acetylated on endogenous MeCP2. Acetylation of MeCP2 detected by Western blotting. (A) PC3 cells were treated with deacetylase inhibitors: DMSO as vehicle control, and SIRT1/2 Inhibitor-IV for a short time period range from 10 min to 1:15 h. (B) MDA-MB-468 were treated with deacetylase inhibitors: DMSO as vehicle control, and SIRT1/2 Inhibitor-IV for a short time period range from 10 to 120 min. (C) MDA-MB-468 were treated with deacetylase inhibitors: DMSO as vehicle control, and with various doses of SIRT1/2 Inhibitor-IV. For all immunoprecipitations equal amount of protein were loaded for each immunoprecipitation set up using acetyl-lysine (Ac-K) antibody as per protocol. Acetylation of MeCP2 was detected by Western blotting along with positive control, whole cell extract (WCE) using MeCP2 specific antibody. Species-matched IgG was used as a negative control. IgG heavy chain (IgG Hc) was blotted for as a control for equal antibody loading for immunoprecipitation and GAPDH for WCE. (D) The table indicates putative lysine residues that were found to be acetylated on MeCP2 under basal condition (DMSO) and upon deacetylase inhibition using 2 µM panobinostat (PANO), 10 µM Inhibitor-IV (IV), 10 µM Inhibitor-VII (VII), and 10 µM pracinostat (PRAC) and showed ion peaks at mass/charge (m/z) ratio of ∼126 in PC3 and MDA-MB-468 cells. [file Image_3.tif]

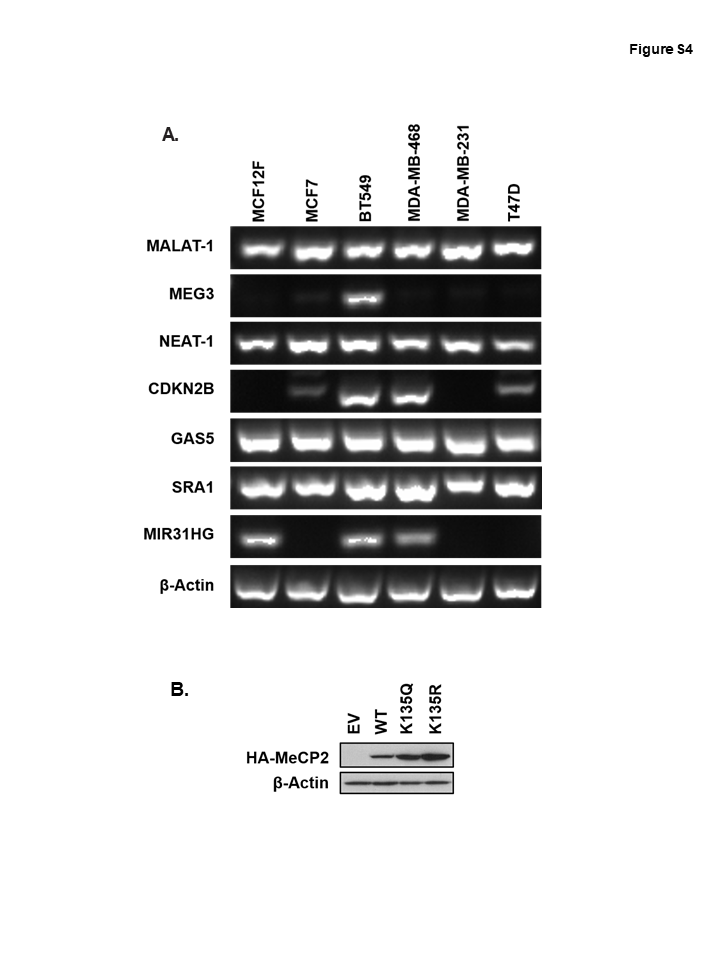

Supplement: Supplementary Figure 4 — Expression profile of lncRNA across normal and breast cancer cell lines. (A) RNA samples were extracted and converted to cDNA by reverse transcriptase enzyme. RT-PCR was performed to determine the expression of MALAT-1, MEG3, NEAT-1, CDKN2B, GAS5, SRA1, MIR31HG LncRNAs, and Beta actin as positive control in MCF12F normal breast cells and MCF7, BT549, MDA-MB-468, MDA-MB-231 and T47D breast cancer cell lines. (B) Stable expression of empty vector (EV), HA-epitope tagged MeCP2 wild type (WT), HA-tagged deacetylation mutants (K to R), HA-tagged acetylation mutants (K to Q) on K135 lysine residues in MDA-MB-468 cells. [file Image_4.tif]
